# Effect of Soybean and Soybean *Koji* on Obesity and Dyslipidemia in Rats Fed a High-Fat Diet: A Comparative Study

**DOI:** 10.3390/ijerph18116032

**Published:** 2021-06-04

**Authors:** Sihoon Park, Jae-Joon Lee, Hye-Won Shin, Sunyoon Jung, Jung-Heun Ha

**Affiliations:** 1 Department of Food and Nutrition, Chosun University, Gwangju 61452, Korea; sihun6312@naver.com (S.P.); leejj80@chosun.ac.kr (J.-J.L.); 2 Food R&D Institute, CJ CheilJedang Corp., Suwon 16495, Korea; hyewon.shin@cj.net; 3 Research Center for Industrialization of Natural Neutralization, Dankook University, Cheonan 31116, Korea; 4 Department of Food Science and Nutrition, Dankook University, Cheonan 31116, Korea

**Keywords:** soybean *koji*, high-fat diet, obesity, dyslipidemia, rats

## Abstract

Soybean *koji* refers to steamed soybeans inoculated with microbial species. Soybean fermentation improves the health benefits of soybeans. Obesity is a serious health concern owing to its increasing incidence rate and high association with other metabolic diseases. Therefore, we investigated the effects of soybean and soybean *koji* on high-fat diet-induced obesity in rats. Five-week-old male Sprague-Dawley rats were randomly divided into four groups (*n* = 8/group) as follows: (1) regular diet (RD), (2) high-fat diet (HFD), (3) HFD + steamed soybean (HFD+SS), and (4) HFD + soybean *koji* (HFD+SK). SK contained more free amino acids and unsaturated fatty acids than SS. In a rat model of obesity, SK consumption significantly alleviated the increase in weight of white adipose tissue and mRNA expression of lipogenic genes, whereas SS consumption did not. Both SS and SK reduced serum triglyceride, total cholesterol, and low-density lipoprotein cholesterol levels, and increased high-density lipoprotein cholesterol levels. SS and SK also inhibited lipid accumulation in the liver and white adipose tissue and reduced adipocyte size. Although both SS and SK could alleviate HFD-induced dyslipidemia, SK has better anti-obesity effects than SS by regulating lipogenesis. Overall, SK is an excellent functional food that may prevent obesity.

## 1. Introduction

Soybeans are an important dietary source because they contain high-quality proteins and are rich in bioactive compounds such as isoflavones and saponins [1]. Nowadays, diet supplementation has been widely accepted as a useful strategy to modulate biochemical and molecular pathways to improve physiological and pathological conditions [2,3]. It has been reported that soybeans have many therapeutic properties, including hypocholesterolemic [4], anti-hypertensive [5], and anti-cancer effects [6]. In Asia, soybeans are primarily fermented dietary sources to give them a unique flavor and improve their shelf life. Fermented soybeans are used to produce soybean sauces, such as *doenjang*, *gochujang*, *mirin*, sake, and miso. In recent years, fermented foods have gained attention because fermentation can boost the bioactive components responsible for health benefits [7]. Commercially available fermented soybean products use soybean *koji* as a starter in soybean fermentation to speed up the process and ensure food safety [8]. Soybean *koji* is made by steaming soybeans and then inoculating molds or bacteria that are recognized as safe for consumption. Soybean *koji* has an abundance of hydrolytic enzymes that break down soybeans and synthesize new components during fermentation [9]. Previous studies have reported that soybean *koji* is a potent bioactive food material, as it exerts better antioxidant activity than soybeans [6].

Obesity refers to the accumulation of excess body fat due to an energy imbalance. When energy intake exceeds energy expenditure, the surplus energy is stored in the body, leading to adipose tissue enlargement, dyslipidemia, and fatty liver, and is linked to other metabolic diseases such as diabetes mellitus, cardiovascular disease (CVD), and certain types of cancer [10,11]. In modern society, the rate of obesity is increasing due to energy-dense foods and a sedentary lifestyle [12]. According to the National Health and Nutrition Examination Survey (NHANES), the rate of severe obesity in the US increased from 4.7% to 9.2% during 1999–2000 and 2015–2016, respectively [13]. More recently, it has been reported that people are more likely to become obese due to restrictions on physical activity caused by the COVID-19 pandemic [14]. Zhu et al. reported that during the COVID-19 outbreak people spent most of their time at home, resulting in decreased physical activity and increased food intake, which was closely related to weight gain [15]. Obesity is a modifiable factor by regulating food consumption as well as physical activity; therefore, dietary intervention holds more weight for preventing/overcoming obesity when people reduce their physical activity, such as during the COVID-19 lockdown. We may also consider therapeutic regulations in obesity. To date, five drugs have been approved by the US Food and Drug Administration (FDA) for the treatment of obesity, which act as appetite suppressants, metabolic stimulants, or nutrient absorption inhibitors primarily through hormonal action [16]. The aforementioned therapies have significant limitations owing to side effects and weight-loss efficacy. Therefore, dietary and lifestyle interventions are fundamental to countering obesity. As a result, natural materials, including red pepper [17], ginger [18], and green tea [19], which have been reported to possess anti-obesity effects, are preferred for preventing obesity [20].

In previous studies, soy protein has been reported to affect obesity by lowering body weight, fasting glucose levels, and hepatic fat accumulation in animal models [21]. Soybean polysaccharides and genistein prevent high fat-induced body weight gain, dyslipidemia, oxidative stress, and inflammation in mice [22]. However, the effects of fermented soybeans on obesity are not fully understood yet. Recently, Kim et al. developed soybean *koji* inoculated with *Bacillus amyloliquefaciens* CJ 14-6, which has excellent protease and amylase activities [23]. *Gochujang*, a typical traditional Korean sauce produced using the soybean *koji*, showed an anti-obesity effect [24]; however, the effect of soybean *koji* on metabolic alterations induced by a high-fat diet has not yet been elucidated. Therefore, this study aimed to investigate and compare the effects of soybean and soybean *koji* inoculated with *Bacillus amyloliquefaciens* CJ 14-6 on high-fat diet-induced obesity and obesity-induced metabolic changes in rats, and to evaluate whether soybean *koji* could be used as a better alternative to prevent obesity.

## 2. Materials and Methods

### 2.1. Preparation of Steamed Soybean (SS) and Soybean Koji (SK)

The steamed soybean (SS) and soybean *koji* (SK) used in this study were supplied by the CJ CheilJedang Corporation (Suwon, South Korea). For the preparation of SS, soybeans were immersed in water at 15 °C for 15 h and then steamed at 40 °C for 30 min. After cooling and drying at room temperature (RT; 20–25 °C) for 24 h, the soybeans were ground into powder. *Bacillus amyloliquefaciens* CJ 14-6 is a patented strain of the CJ CheilJedang Corporation (KCCM 11718P). To obtain SK, *Bacillus amyloliquefaciens* CJ 14-6 was cultured at 37 °C for 24 h on a rotary shaker at 200 rpm. Then, 2% (*v/w*) of the culture solution, based on the weight of the soybeans, was inoculated into the steamed soybeans and incubated at 37 °C for 36 h. The fermented soybeans were then dried at 40 °C for 24 h and pulverized into a powder.

### 2.2. Proximate Composition of SS and SK

The general composition analysis of SS and SK was carried out in accordance with the Association of Official Analytical Chemists method [18]. Moisture content was determined using the oven-drying method at 105 °C. Crude protein content was determined using the micro-Kjeldahl method. The crude fat content was analyzed using a Soxhlet apparatus. The ash content was analyzed by ignition in an electric furnace. Carbohydrates were calculated by subtracting the moisture, crude protein, crude fat, and ash contents from 100. The fiber content was determined using an enzymatic-gravimetric method. All analyses were performed in triplicate.

### 2.3. Free Amino Acid Content of SS and SK

Free amino acid analysis was conducted by hydrolyzing samples (0.5 g) with 3 mL of 6 N HCl at 121 °C for 24 h. Excess acid was removed using a rotary vacuum evaporator, and the sample was then dissolved in 10 mL of sodium phosphate buffer (pH 7.0) for further analysis [25]. The solution (1 mL) was filtered through a membrane filter (0.2 μm), and amino acid analysis was carried out in a Biochrome 20 Amino Acid Analyzer (Pharmacia Biotech, Cambridge, UK). All analyses were performed in triplicate.

### 2.4. Fatty Acid Content of SS and SK

Fatty acid analysis was conducted following the Wijngaarden method [26]. The lipids in the samples (2 g) were extracted with ether, filtered, and concentrated under reduced pressure. Lipid samples (100 mg) were transferred to Erlenmeyer flasks and stirred with 4 mL of 1 N KOH ethanol until the lipid droplets disappeared. Following the addition of 5 mL of 14% BF_3_-methanol, the samples were heated at 80 °C for 5 min to synthesize methyl ester. The samples were cooled again; 3 mL of saturated NaCl solution and 1 mL of hexane were added and allowed to stand to separate the solution. The supernatant was transferred to a new tube and mixed with anhydrous Na_2_SO to remove moisture. The fatty acids in the solution were analyzed using gas chromatography (GC-10A, Shimadzu, Kyoto, Japan). All analyses were performed in triplicate.

### 2.5. Animal Experiments and Diets

All animal studies were approved by the Chosun University Institutional Animal Care and Use Committee (C IACUC No. 2015-A0028). After 1 week of acclimatization, 5-week-old male Sprague-Dawley rats (Orient Bio, Inc., Seongnam, Korea) were housed in cages for 8 weeks until sacrifice. The animals had access to food and water *ad libitum* throughout the experimental period. The experimental rats in each group (*n* = 8) were fed one of the following dietary compositions: (1) a regular diet (RD) based on the AIN-93G formulation (15.8% of energy from dietary fat), (2) a high-fat diet (HFD, 39.5% of energy from dietary fat), (3) an HFD diet mixed with 3% SS (HFD+SS, 39.2% of energy from dietary fat), or (4) an HFD diet mixed with 3% SK (HFD+SK, 39.2% of energy from dietary fat). Individual dietary compositions are listed in Table 1. Body weight (BW) and food intake were measured weekly. The food efficiency ratio (FER) was calculated by dividing the total BW gain by total food intake. At the end of the study, final body weights were measured after overnight fasting, and whole blood samples were collected from the heart and the serum was isolated after clotting procedures. The organs were harvested, weighed, and snap-frozen in liquid nitrogen after being sacrificed by thoracotomy after CO_2_ narcosis. Isolated serum and organ samples were stored at −80 °C until further analysis.

### 2.6. Biochemical and Enzymatic Analysis of Serum Samples

The enzymatic activities of alanine aminotransferase (ALT), aspartate aminotransferase (AST), alkaline phosphatase (ALP), lactate dehydrogenase (LDH), triglyceride (TG), total cholesterol (TC), high-density lipoprotein cholesterol (HDL-C), and fasting glucose (GLU) levels were measured using a chemistry analyzer (Fujifilm Dri-C hem 3500i, Fujifilm, Tokyo, Japan) as previously described [27]. The values of low-density lipoprotein cholesterol (LDL-C), atherogenic index (AI), and cardiac risk factor (CRF) were also calculated as previously described [28,29].

### 2.7. Tissue and Fecal Lipid Contents

Lipids were extracted from ~0.1 g of liver and adipose tissues as previously described [30]. The TG and TC levels were measured from the lower (lipid-abundant) layer following previously described methods [31,32].

### 2.8. Reverse Transcription-Polymerase Chain Reaction (RT-PCR)

Total RNA was extracted from the samples using NucleoSpin RNA Plus (Macherey-Nagel GmbH & Co., Düren, Germany) according to the manufacturer’s protocol. RT-PCR was performed as described previously [33]. The expression of each experimental gene was normalized to the expression of *β-actin*, which did not significantly vary between the different dietary settings. The gene-specific oligonucleotide primers used in this study are listed in Table 2.

### 2.9. Histological Analysis

Liver and epididymal adipose tissues were fixed, sectioned, and stained as previously described [27].

### 2.10. Statistical Analysis

The experimental data were analyzed using one-way analysis of variance (ANOVA; GraphPad PRISM 8, San Diego, CA, USA). Subsequently, Tukey’s post-hoc test was applied to distinguish groups that varied significantly (*p* < 0.05).

## 3. Results

### 3.1. Proximate, Free Amino Acid, and Fatty Acid Compositions in Steamed Soybean and Soybean Koji

To investigate the changes in the composition of soybeans following fermentation, the proximate composition and free amino acid and fatty acid contents in SS and SK were analyzed. As shown in Table 3, SK contained more moisture and crude fat than SS; however, the carbohydrate content in SS was higher than that in SK. On the other hand, the crude protein, ash, and dietary fiber contents did not significantly differ between SS and SK.

Free amino acid analysis of SS and SK revealed 23 amino acids (Table 4). The total free amino acid content in SK was 4.3-fold higher than that in SS (*p* < 0.05). Specifically, the contents of urea, threonine, glutamic acid, glycine, valine, isoleucine, leucine, tyrosine, phenylalanine, histidine, and tryptophan were higher in SK than in SS (*p* < 0.05), and the difference in the contents of leucine and phenylalanine was the largest.

The fatty acid compositions of SS and SK are listed in Table 5. SS contained more saturated fatty acids than SK, whereas SK had more monounsaturated fatty acids (MUFA) and polyunsaturated fatty acids (PUFA) (*p* < 0.05). The predominant components of SS were linoleic acid (C18:2n6c), heneicosanoic acid (C21:0), oleic acid (C18:1n9c), and palmitic acid (C16:0). Similarly, linoleic acid (C18:2n6c), oleic acid (C18:1n9c), palmitic acid (C16:0), and heneicosanoic acid (C21:0) were the most abundant fatty acids in SK. Compared to those in SS, the amounts of linolenic acid (C18:3n3), stearic acid (C18:0), linoleic acid (C18:2n6c), and oleic acid (C18:1n9c) were significantly higher in SK (*p* < 0.05).

### 3.2. SS and SK Consumption Tended to Ameliorate HFD-Induced Body Weight Gain

**Figure 1:**
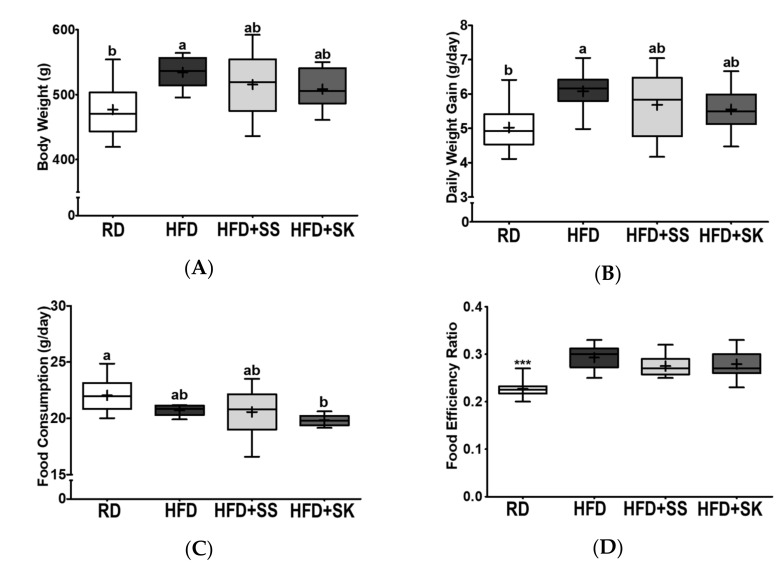
Effects of steamed soybean (SS) and soybean *koji* (SK) on final body weight, daily body weight gain, daily average food consumption, and food efficiency ratio in HFD-fed Sprague-Dawley rats. Experimental rats were fed a regular diet (RD) or a high-fat diet (HFD) with 3% SS (HFD+SS) or with 3% SK (HFD+SK) for 8 weeks. (**A**) The final body weight, (**B**) daily body weight gain ((the final BW after dietary feeding—the initial BW)/day), (**C**) daily average food consumption, and (**D**) food efficiency ratio were measured or calculated. RD, regular diet; HFD, high-fat diet; HFD+SS, high-fat diet + 3% steamed soybean; HFD+SK, high-fat diet + 3% steamed soybean. Values are displayed as a box-and-whisker plot with means (expressed as ‘+’), *n* = 8. Data were analyzed using one-way ANOVA followed by Tukey’s post-hoc comparison. Means labeled without a common letter differ significantly, * *p* < 0.05. *** *p* < 0.001.

At the end of the experiment, the body weight and daily weight gain of the HFD group were significantly higher than those of the RD group (*p* < 0.05) (Figure 1A,B). The body weight and daily weight gain of rats supplemented with SS or SK tended to be lower than those of the HFD group; however, the differences were not statistically significant (Figure 1A,B).

To determine whether the amount of food intake was associated with changes in the body weight, daily food consumption and FER were measured. Daily food consumption of the HFD group was slightly lower than that of the RD group, but there was no statistical difference; nevertheless, rats fed HFD with SK showed significantly lower food consumption compared to the RD group (*p* < 0.05) (Figure 1C). The FER, determined by dividing the body weight gain by the amount of food consumed, was significantly higher in the HFD group than in the RD group (*p* < 0.05). SS and SK did not affect the FER increased by HFD, indicating that weight changes between HFD and SS- and SK-supplemented groups were related to the alterations in food intake (Figure 1D).

### 3.3. SK Consumption Ameliorated HFD-Induced Fat Accumulation in White Adipose Tissue

**Figure 2:**
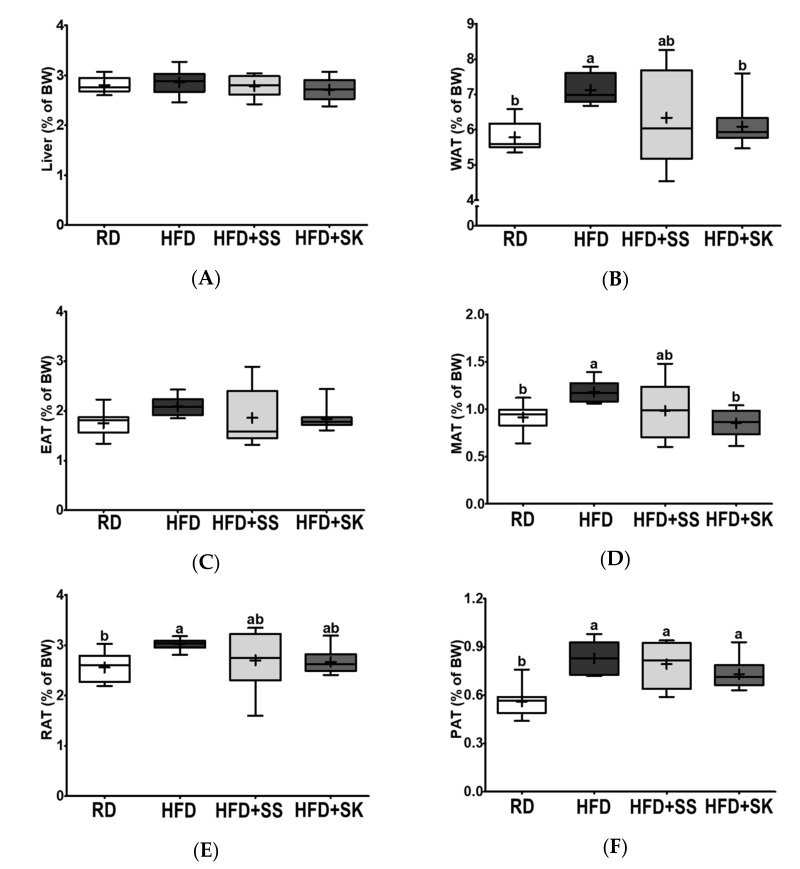
Effects of steamed soybean (SS) and soybean *koji* (SK) on the relative liver and white adipose tissue weight in HFD-fed Sprague-Dawley rats. Experimental rats were fed a regular diet (RD) or a high-fat diet (HFD) with 3% SS (HFD+SS) or with 3% SK (HFD+SK) for 8 weeks. Relative weights of (**A**) liver, (**B**) white adipose tissue (WAT), (**C**) epididymal adipose tissue (EAT), (**D**) mesenteric adipose tissue (MAT), (**E**) retroperitoneal adipose tissue (RAT), and (**F**) perirenal adipose tissue (PAT). Relative tissue weights (%) were calculated as organ weight (g)/final BW × 100. RD, regular diet; HFD, high-fat diet; HFD+SS, high-fat diet + 3% steamed soybean; HFD+SK, high-fat diet + 3% steamed soybean. Values are displayed as a box-and-whisker plot with means (expressed as ‘+’), *n* = 8. Data were analyzed using one-way ANOVA followed by Tukey’s post-hoc comparison. ^a,b^ Means labeled without a common letter differ significantly, *p* < 0.05.

As the liver and adipose tissue have central roles in regulating whole-body metabolism, we measured the weights of the liver and white adipose tissue fat pads. The liver weights of the rats were not statistically different among the four groups (Figure 2A). White adipose tissue (WAT) weight was significantly higher in the HFD group than in the RD group. In contrast, SK significantly inhibited HFD-induced WAT accumulation (*p* < 0.05) (Figure 2B). The weight of epididymal adipose tissue (EAT) did not significantly differ among the groups; however, mesenteric adipose tissue (MAT), retroperitoneal adipose tissue (RAT), and perirenal adipose tissue (PAT) weights were significantly higher in the HFD group than in the RD group (*p* < 0.05) (Figure 2C–F). Specifically, the weight of MAT was lower in HFD+SK group than in the HFD group (*p* < 0.05) (Figure 2D).

### 3.4. SS and SK Consumption Improved the Levels of Serum Metabolites Related to Liver Function and Lowered Leptin Level

**Figure 3:**
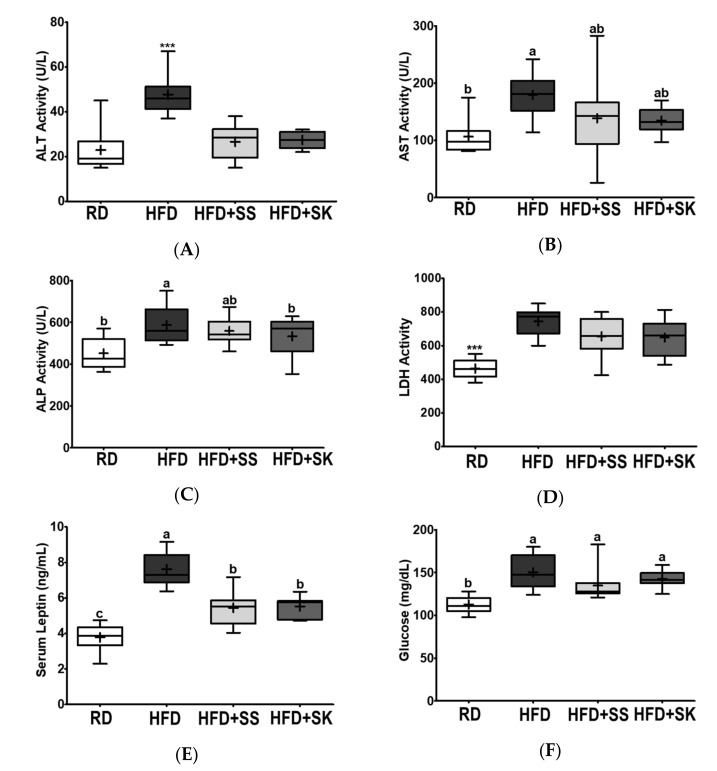
Effect of steamed soybean (SS) and soybean *koji* (SK) on biochemical markers of hepatic function, leptin, and glucose in HFD fed Sprague-Dawley rats. Experimental rats were fed a regular diet (RD) or a high-fat diet (HFD) with 3% SS (HFD+SS) or with 3% SK (HFD+SK) for 8 weeks. (**A**) Alanine aminotransferase (ALT), (**B**) aspartate aminotransferase (AST), (**C**) alkaline phosphatase (ALP), and (**D**) lactate dehydrogenase (LDH) activities and (**E**) leptin and (**F**) fasting glucose levels were analyzed enzymatically or biochemically. RD, regular diet; HFD, high-fat diet; HFD+SS, high-fat diet + 3% steamed soybean; HFD+SK, high-fat diet + 3% steamed soybean. Values are displayed as a box-and-whisker plot with means (expressed as ‘+’), *n* = 8. Data were analyzed using one-way ANOVA followed by Tukey’s post-hoc comparison. Means labeled without a common letter differ significantly, *p* < 0.05. *** *p* < 0.001.

Serum ALT, AST, ALP, and LDH activities are typical indicators of liver function [34]. Leptin, an adipokine that exerts endocrine function, regulates metabolic homeostasis by regulating appetite, energy expenditure, and glucose utilization [35]. In this study, HFD increased the serum ALT, AST, ALP, and LDH activities compared to the RD (*p* < 0.05) (Figure 3A–D). However, rats that consumed the HFD+SS and HFD+SK diets had lower serum ALT activities than those in the HFD group (*p* < 0.05) (Figure 3A). Additionally, ALP activity was significantly lower in the HFD+SK group than in the HFD group (*p* < 0.05) (Figure 3C).

Serum leptin levels in the HFD group were significantly higher than those in the RD group; however, SS and SK inhibited HFD-induced increase in serum leptin levels (*p* < 0.05) (Figure 3E). The HFD group had a higher fasting serum glucose level than the RD group (*p* < 0.05), and the levels in the HFD+SS and HFD+SK groups did not significantly differ from those in the HFD group (Figure 3F).

### 3.5. SS and SK Consumption Improved the Serum Lipid Profile

**Figure 4:**
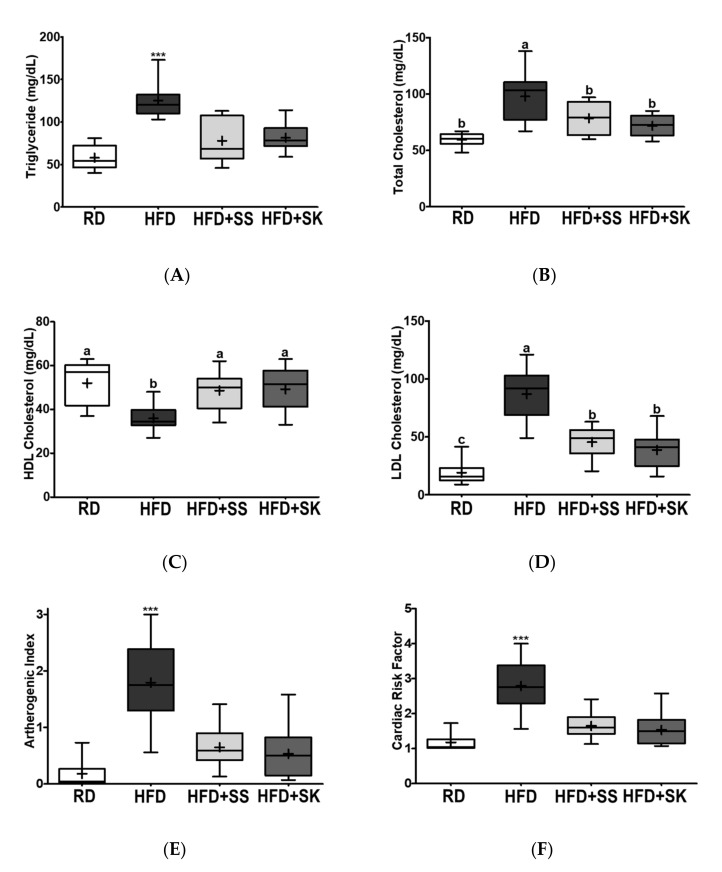
Effect of steamed soybean (SS) and soybean *koji* (SK) on lipid panels, atherogenic index, and cardiovascular disease risk factors. Experimental rats were fed a regular diet (RD) or a high-fat diet (HFD) with 3% SS (HFD+SS) or with 3% SK (HFD+SK) for 8 weeks. Serum levels of (**A**) triglyceride (TG), (**B**) total cholesterol (TC), and (**C**) high-density lipoprotein-cholesterol (HDL-C) were measured in the experimental rats. (**D**) Low-density lipoprotein-cholesterol (LDL-C), (**E**) atherogenic index (AI), and (**F**) cardiac risk factor (CRF) were processed. RD, regular diet; HFD, high-fat diet; HFD+SS, high-fat diet + 3% steamed soybean; HFD+SK, high-fat diet + 3% steamed soybean. Values are displayed as a box-and-whisker plot with means (expressed as ‘+’), *n* = 8. Data were analyzed using one-way ANOVA followed by Tukey’s post-hoc comparison. Means labeled without a common letter differ significantly, *p* < 0.05. *** *p* < 0.001.

Serum lipid levels are one of the most important predictors of metabolic disease. High LDL-C and low HDL-C levels are highly correlated with the incidence of CVD. In this study, the HFD group had higher serum TG, TC, and LDL-C and lower HDL-C levels than the RD group (*p* < 0.05) (Figure 4A–D). On the other hand, SS and SK significantly inhibited the HFD-induced increase in serum TG, TC, and LDL-C and the reduction in HDL-C (*p* < 0.05) (Figure 4A–D). Consequently, SS and SK significantly inhibited the increase in the HFD-induced atherogenic index and cardiac risk factor (*p* < 0.05) (Figure 4E,F).

### 3.6. SS and SK Consumption Inhibited HFD-Induced Hepatic Lipid Accumulation

**Figure 5:**
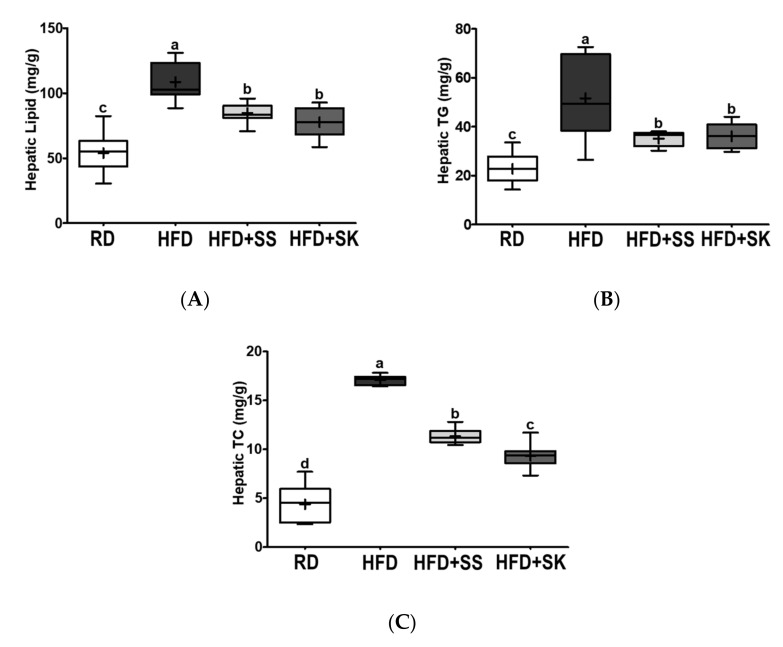

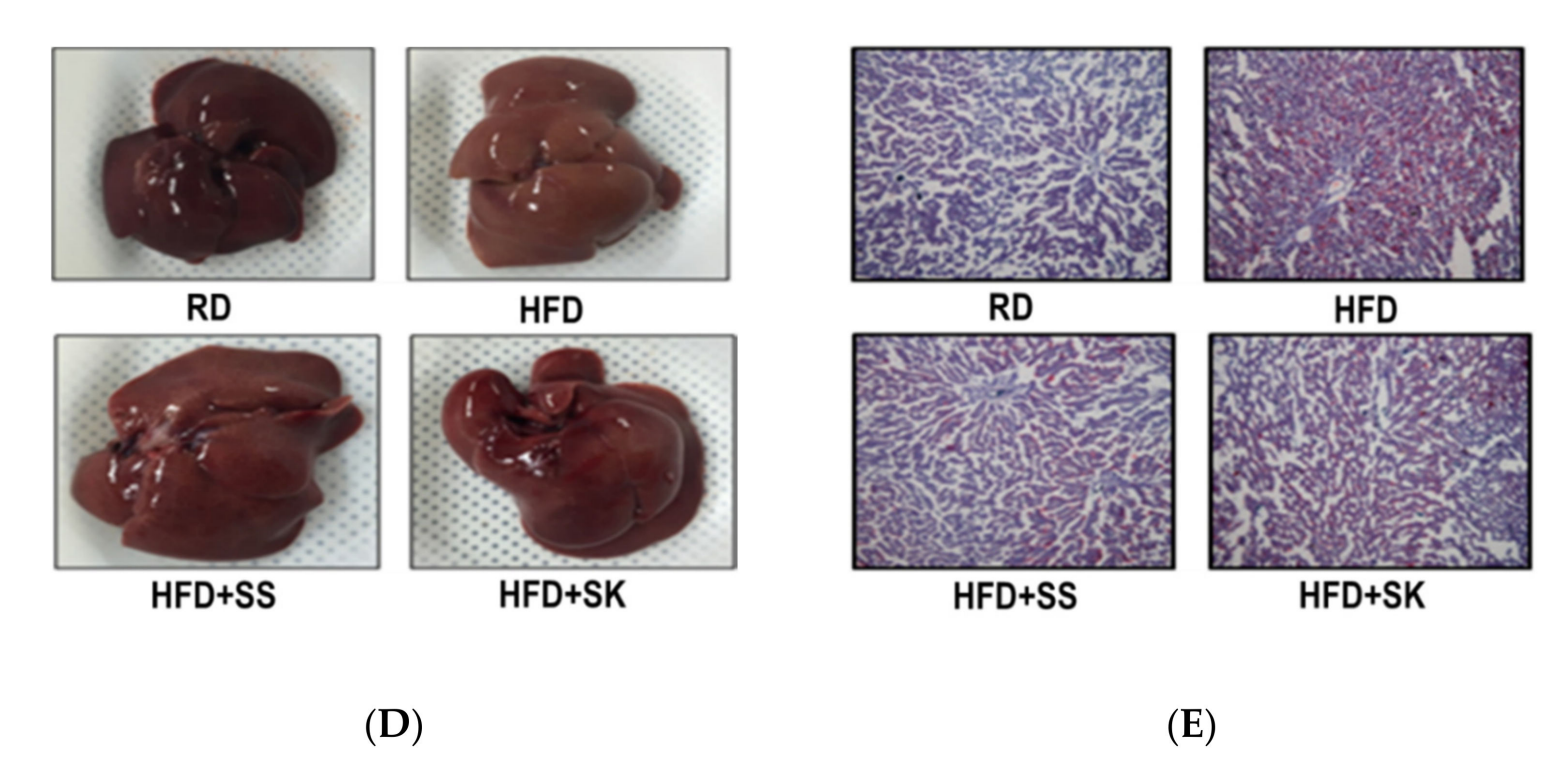
Effect of steamed soybean (SS) and soybean *koji* (SK) on fat accumulation in the liver. Experimental rats were fed a regular diet (RD) or a high-fat diet (HFD) with 3% SS (HFD+SS) or with 3% SK (HFD+SK) for 8 weeks. (**A**) Hepatic lipid, (**B**) hepatic triglyceride (TG), and (**C**) hepatic total cholesterol (TC) were measured and normalized by the measured tissue weight (g). (**D**) Harvested whole liver and (**E**) liver samples fixed and stained with Oil red O staining from each experimental group. RD, regular diet; HFD, high-fat diet; HFD+SS, high-fat diet + 3% steamed soybean; HFD+SK, high-fat diet + 3% steamed soybean. Values are displayed as a box-and-whisker plot with means (expressed as ‘+’), *n* = 8. Data were analyzed using one-way ANOVA followed by Tukey’s post-hoc comparison. Means labeled without a common letter differ significantly, *p* < 0.05.

The liver metabolizes most nutrients, and chronic HFD consumption can lead to fatty liver due to lipid overload. Therefore, we measured the levels of total lipid, TG, and TC in the liver to investigate whether SS and SK had a beneficial effect on hepatic lipid accumulation induced by HFD. As shown in Figure 5A, the HFD group had a higher hepatic lipid level than the RD group (*p* < 0.05). The hepatic levels of TG and TC were also significantly higher in the HFD group than in the RD group (*p* < 0.05) (Figure 5B,C). Rats that consumed SS and SK had lower total lipid, TG, and TC levels in the liver compared to those in the HFD group (*p* < 0.05) (Figure 5A–C). Moreover, hepatic TC levels were significantly lower in rats fed with SK than in rats fed with SS (*p* < 0.05) (Figure 5C).

Figure 5D shows the representative liver images of each group. The liver of the HFD group appeared brighter than that of the RD group; however, SS and SK ameliorated the morphological changes caused by HFD (Figure 5D). Similarly, Oil Red O-stained images of the liver sections showed that HFD increased lipid accumulation in the liver. In contrast, SS and SK improved hepatic lipid accumulation induced by HFD (Figure 5E).

### 3.7. SS and SK Consumption Inhibited HFD-Induced Lipid Accumulation in White Adipose Tissue

**Figure 6:**
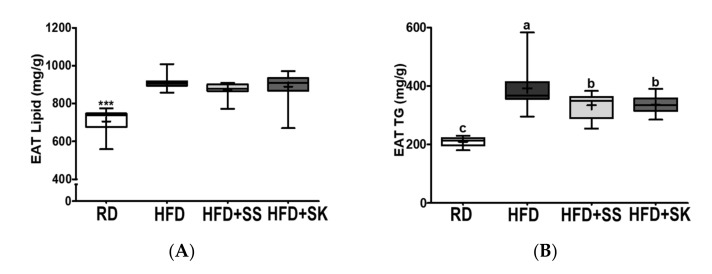

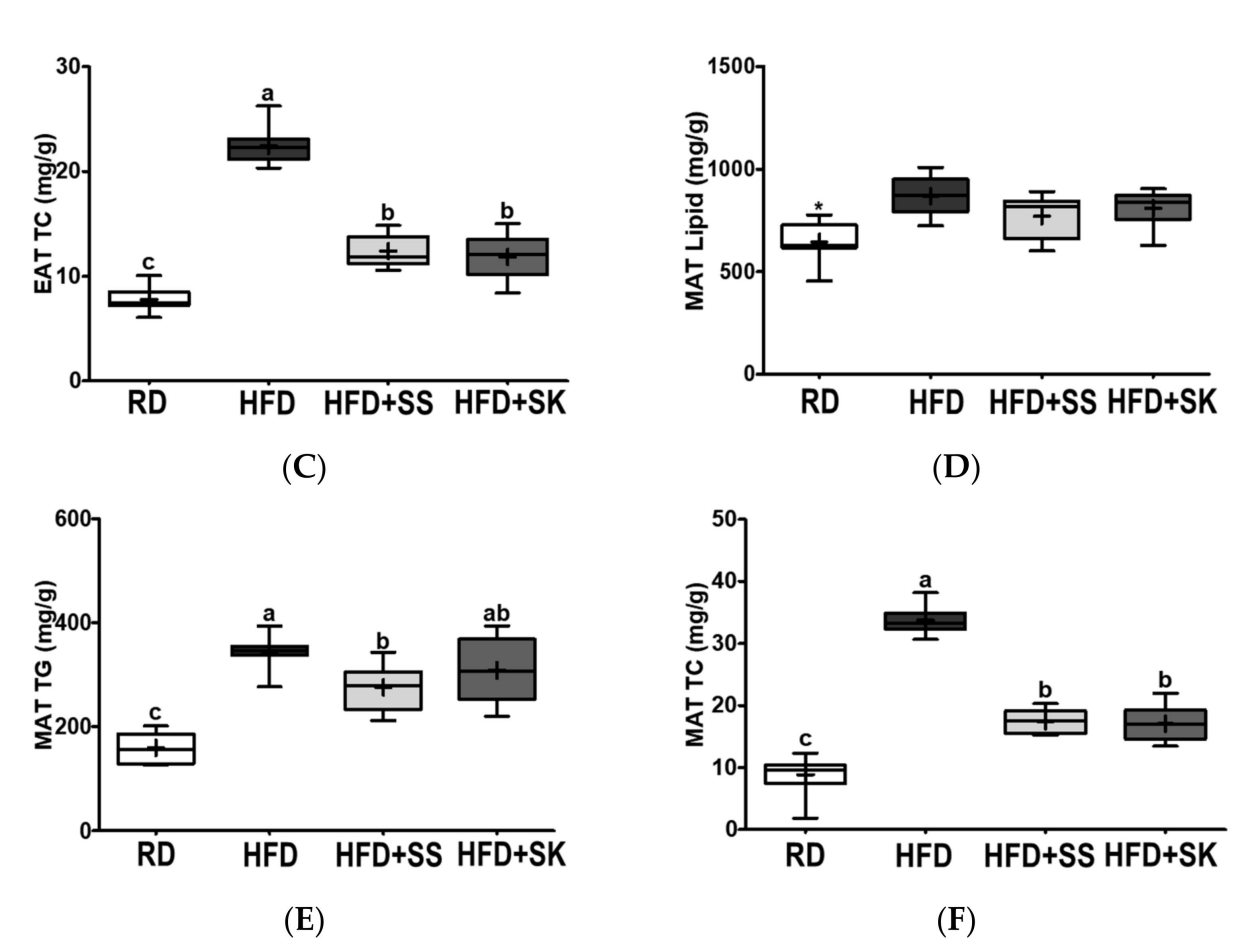
Effect of steamed soybean (SS) and soybean *koji* (SK) on lipid, triglyceride (TG), and total cholesterol (TC) deposition in the epididymal adipose tissue (EAT) and mesenteric adipose tissue (MAT). Experimental rats were fed a regular diet (RD) or a high-fat diet (HFD) with 3% SS (HFD+SS) or with 3% SK (HFD+SK) for 8 weeks. (**A**,**D**) Lipid, (**B**,**E**) TG, and (**C**,**F**) TC were measured from the epididymal adipose tissue (EAT) and mesenteric adipose tissue (MAT) and normalized by the measured tissue weight (g). RD, regular diet; HFD, high-fat diet; HFD+SS, high-fat diet + 3% steamed soybean; HFD+SK, high-fat diet + 3% steamed soybean. Values are displayed as a box-and-whisker plot with means (expressed as ‘+’), *n* = 8. Data were analyzed using one-way ANOVA followed by Tukey’s post-hoc comparison. Means labeled without a common letter differ significantly, *p* < 0.05.

Adipose tissue is a central organ that accumulates excessive energy in the form of triglycerides (TG). Therefore, we analyzed the levels of total lipids, TG, and TC in WAT. We also measured adipocyte size from EAT, because lipid accumulation contributes to adipocyte hypertrophy. In EAT, the HFD group had higher total lipid, TG, and TC levels than the RD group (*p* < 0.05). In contrast, SS and SK significantly inhibited TG and TC accumulation (*p* < 0.05) (Figure 6A–C). In MAT, total lipid, TG, and TC levels were also significantly higher in the HFD group than in the RD group (*p* < 0.05); however, the TG level in the HFD+SS group and the TC levels in the HFD+SS and HFD+SK groups were significantly lower than those in the HFD group (*p* < 0.05) (Figure 6D–F).

**Figure 7:**
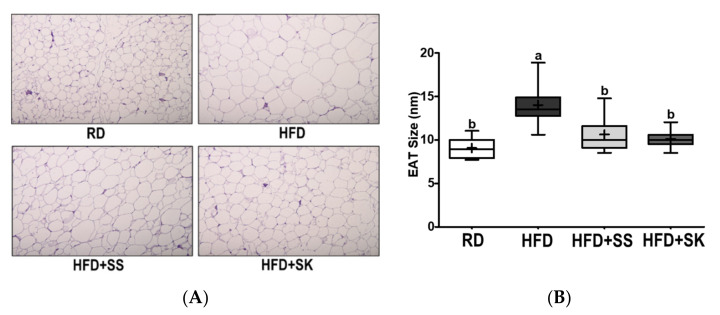
Effect of steamed soybean (SS) and soybean *koji* (SK) on epididymal adipocyte size. Experimental rats were fed a regular diet (RD) or a high-fat diet (HFD) with 3% SS (HFD+SS) or with 3% SK (HFD+SK) for 8 weeks. The epididymal adipose tissue (EAT) was stained with hematoxylin and eosin (HE). Magnification, 100×. The EAT surface area was quantified using the Image J program. (**A**) Representative EAT images. (**B**) Quantification of the surface area of EAT. RD, regular diet; HFD, high-fat diet; HFD+SS, high-fat diet + 3% steamed soybean; HFD+SK, high-fat diet + 3% steamed soybean. Values are displayed as a box-and-whisker plot with means (expressed as ‘+’), n = 8. Data were analyzed using one-way ANOVA followed by Tukey’s post-hoc comparison. Means labeled without a common letter differ significantly, *p* < 0.05.

Figure 7A shows the H&E-stained images of the EAT sections. The adipocyte size of the HFD group was greater than that of the RD group; however, SS and SK appeared to ameliorate adipocyte hypertrophy induced by HFD. Consistently, the adipocyte size in the HFD group was significantly larger than that in the RD group; however, the adipocyte size was reduced considerably by SS and SK consumption (*p* < 0.05) (Figure 7B).

### 3.8. SS and SK Consumption Suppressed mRNA Expression Involved in Lipogenesis in EAT

**Figure 8:**
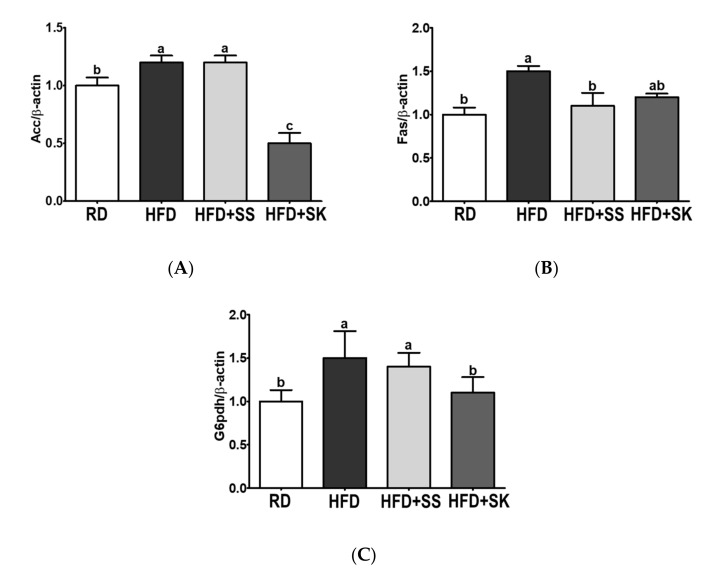
Effect of steamed soybean (SS) and soybean *koji* (SK) on epididymal adipocyte mRNA expression. Experimental rats were fed a regular diet (RD) or a high-fat diet (HFD) with 3% SS (HFD+SS) or with 3% SK (HFD+SK) for 8 weeks. (**A**) Acetyl-CoA carboxylase (*Acc*), (**B**) fatty acid synthase (*Fas*), and (**C**) glucose-6-phosphate dehydrogenase (*G6pdh*) mRNA expression was analyzed using qRT-PCR and normalized by β-actin. RD, regular diet; HFD, high-fat diet; HFD+SS, high-fat diet + 3% steamed soybean; HFD+SK, high-fat diet + 3% steamed soybean. Values are means ± standard deviation, n = 8. Data were analyzed using one-way ANOVA followed by Tukey’s post-hoc test. Means labeled without a common letter differ significantly, *p* < 0.05.

To examine the possible mechanism by which SS and SK ameliorate obesity, we measured the mRNA expression levels in epididymal adipose tissue. Acetyl-CoA carboxylase (ACC), fatty acid synthase (FAS), and glucose-6-phosphate dehydrogenase (G6PDH) are vital enzymes that regulate lipid synthesis. In this study, the mRNA expression of *Acc*, *Fas*, and *G6pdh* was higher in the HFD group than in the RD group (*p* < 0.05) (Figure 8A–C). On the other hand, the mRNA levels of *Acc* and *G6pdh* were significantly lower in the HFD+SK group than in the HFD group (*p* < 0.05) (Figure 8A,C). The *Fas* mRNA levels were also lower in the HFD+SS and HFD+SK groups than in the HFD group, but a significant difference was observed only in the HFD+SS group (*p* < 0.05) (Figure 8B).

## 4. Discussion

Soybean fermentation improves the health benefits of soybeans, as fermentation increases the bioactive peptides and free isoflavones [36]. Whereas traditional soybean fermentation relies on spontaneously colonized inoculum, industrial fermentation uses *koji* to maintain consistent quality. *Koji* refers to steamed grains inoculated with well-characterized microbial species under controlled conditions. The anti-obesity effects of soybean protein and soybean polysaccharides have been well documented [22,37]. Moreover, Shin et al. reported that *gochujang*, made using soybean *koji*, alleviates obesity [24], but there is a lack of information on whether soybean *koji* directly influences obesity. Therefore, we investigated whether soybean *koji* prevents high-fat diet-induced obesity and metabolic disorders better than soybean in rats. The present study, for the first time, demonstrated that eight weeks of SK consumption ameliorated HFD-induced obesity more effectively than SS, and both SS and SK have beneficial effects on HFD-induced dyslipidemia in the experimental rats.

The microorganism used in the present study was *Bacillus amyloliquefaciens* CJ 14-6. Bacillus species, including *B. amyloliquefaciens* and *B. subtilis*, have high growth rates and abundant hydrolytic enzymes [38]. According to proximate composition analysis, SK had a higher moisture and crude fat ratio than SS, but the carbohydrate ratio was lower in SK than in SS. Microorganisms produce moisture as a byproduct through respiratory oxidation, using carbohydrates as an energy source. Therefore, the increase in moisture and fat content after fermentation may be due to a decrease in the carbohydrate ratio in the total mass, resulting in a redistribution of nutrient percentages. Nevertheless, the percentage of protein did not increase significantly, which may be due to the partial degradation of proteins during fermentation. Consistently, the free amino acid analysis showed that SK contained more free amino acids, especially leucine and phenylalanine, than SS. Both leucine and phenylalanine are essential amino acids with hydrophobic side chains. Leucine has been reported to inhibit high-fat diet-induced weight gain, hyperglycemia, and hypercholesterolemia in mice [39]. In addition, phenylalanine has been reported to promote cholecystokinin, a satiety hormone that reduces energy intake [40]. It is not clear whether the increased leucine and phenylalanine in this study contributed to metabolic alterations through increased bioavailability in rats. However, previous studies have reported that the requirement and digestibility of leucine and phenylalanine are relatively high compared to other amino acids, suggesting that the beneficial effects of SK may be due in part to the leucine and phenylalanine [41]. Regarding fatty acid composition, SK comprised more MUFA and PUFA than SS; however, the differences were not as significant as those in amino acids. Therefore, we logically postulated that SK might more effectively improve metabolic changes in obesity than SS, which may be related to the enhanced composition of free amino acids.

Excessive energy intake from a high-fat diet is one of the leading causes of obesity in modern society. In a previous in vivo study, chronic high-fat diet consumption induced body weight gain, hepatic steatosis, and insulin resistance [42]. In this study, eight weeks of high-fat diet consumption resulted in a 12.1% increase in body weight compared to RD consumption, despite the indifferent dietary intake. In addition, HFD increased the white adipose tissue weight, the sum of EAT, MAT, RAT, and PAT mass, by 37.9% compared to the RD group, indicating that the majority of body weight gain in the HFD group may be due to the weight increase of WAT. However, SK suppressed the WAT increase caused by a high-fat diet, and most of the reduction was found in MAT. Similar tendencies were observed in the SS group, but the differences were not as significant as those in SK, indicating that SK inhibits obesity more effectively than SS. Although the SK group had lower food consumption than the RD group, the anti-obesity effect of SK may not have been only due to an anorexic effect, because the difference in FER between the HFD and SK groups was not statistically significant. In previous studies, the anti-obesity effect of soy protein was linked to its ability to modulate adipogenic and oxidative capacity and adipokine production [43]. Therefore, we suggest that SK may have protective effects against obesity by partially regulating adipose tissue metabolism.

The liver is the main organ that metabolizes nutrients and detoxifies harmful substances in the body. In obesity, nutrient overload and increased inflammatory mediators hinder hepatic function, thereby increasing the enzymatic activities of AST, ALT, ALP, and LDH [44]. AST and ALT are enzymes that participate in gluconeogenesis by transferring an α-amino group of aspartic acid and alanine to ketoglutaric acid to generate oxalic acid and pyruvic acid [34]. Both AST and ALT are concentrated in the liver, but liver damage eventually increases serum aminotransferase levels [34]. In general, mild aminotransferase elevation is found in non-alcoholic fatty liver disease [45]. ALP is an enzyme involved in the transport of metabolites through the cell membrane. Although elevated ALP levels do not always imply liver damage, it has been reported that the most common cause of elevated ALP levels is liver disease [45]. LDH is present in most living cells, but at higher levels in the muscle, liver, and kidney. When cells are damaged, LDH leaks from the cells into the blood, so elevated levels of LDH indicate some form of organ damage. In the present study, HFD increased AST, ALT, ALP, and LDH activities, similar to a previous study [46], whereas SK alleviated the increase in ALP levels. As ALT is mostly present in the liver, an increase in ALT activity is a more specific indicator of liver damage than other such indicators [34]. Therefore, from these results, we assumed that both SS and SK were not hepatotoxic, but SK was thought to better alleviate the decline in liver function due to obesity.

Leptin is an adipokine secreted from adipose tissue that acts as a hormone through systemic circulation. Leptin was first revealed to regulate appetite, and recently, its regulatory effects on inflammation and blood glucose levels have been reported [47]. In this study, both SS and SK effectively inhibited the HFD-induced increase in serum leptin levels. Since leptin production is related to the size of the adipose tissue [48], the decrease in leptin may be associated with body fat reduction. Furthermore, in this study, we have shown that the HFD+SK group had a lower dietary intake than the RD group, whereas the HFD and HFD+SS groups did not. Therefore, further research on the effect of SK on appetite control is plausible. Previous studies have shown that obesity leads to systemic inflammation through dysregulated adipokine production, resulting in insulin resistance [49]. However, despite the anti-obesity action, the serum glucose increase during HFD feeding was not ameliorated by SK. Therefore, it is assumed that HFD interferes with glucose utilization through other metabolic changes apart from obesity, and SS and SK did not effectively alleviate these pathways.

Obesity is a risk factor for CVD, partly due to impaired lipid homeostasis. The hallmark of dyslipidemia in obesity is increased TG, high LDL-C, and low HDL-C levels [50]. Normally, adipocytes regulate energy homeostasis by storing energy in the form of TG and releasing free fatty acids. However, in an obese state, hypertrophic adipocytes lose their ability to store energy, thus increasing the release and the hepatic influx of fatty acids. Hepatic accumulation of TG increases very-low-density lipoprotein synthesis, which delays the lipolysis of chylomicrons [50]. These TG-rich lipoproteins stimulate TG transfer to LDL and HDL particles, leading to the formation of atherosclerotic LDL particles and decreased HDL-C concentration [51]. In the present study, SS and SK alleviated HFD-induced dyslipidemia by lowering TG, TC, LDL-C, and increasing HDL-C levels. Moreover, SS and SK inhibited hepatic lipid accumulation caused by HFD by reducing TG and TC levels, indicating that both SS and SK may have functions against dyslipidemia and fatty liver in obesity. These results concur with previous studies reporting that soybeans fermented with *Enterococcus faecium* and *Lactobacillus jugurti* reduce cholesterolemia in Wistar rats fed a hypercholesterolemic diet [52]. Anthony et al. reported that isoflavones are responsible for the effects of soy protein on serum lipid profiles during a mild hypercholesterolemic diet [53]. In this study, SK and SS alleviated dyslipidemia and fatty liver, despite increased body fat. Therefore, we suggest that SS and SK may directly regulate lipid metabolism, and further research is required to elucidate the underlying mechanism.

Chronic high-fat consumption increases adipocyte size due to increased fat storage. Therefore, we also measured the adipose tissue lipid concentration and adipocyte size. SS and SK effectively alleviated HFD-induced TG and TC accumulation in adipose tissue. Histological analysis also showed that SS and SK alleviated adipocyte hypertrophy. To maintain lipid homeostasis, adipocytes carry out two reciprocal processes, lipogenesis and lipolysis. ACC, FAS, and G6PDH are critical enzymes involved in lipid synthesis in adipocytes. It has been reported that mRNA expression levels of *Acc*, *Fas*, and *G6pdh* are significantly increased in mouse models of obesity [54,55]. ACC plays a role in the synthesis of malonyl-CoA, a major substrate for long-chain fatty acids [54]. FAS catalyzes the last step in the fatty acid biosynthetic pathway, synthesizing palmitate in the presence of malonyl-CoA and NADPH. G6PDH produces the cellular NADPH required for the biosynthesis of fatty acids [54]. Therefore, we analyzed the mRNA expression of *Acc*, *Fas*, and *G6pdh* to verify the possible mechanism underlying the anti-obesity effect of SK. Consistent with the results on adipose tissue weight, the mRNA expression of *Acc*, *Fas*, and *G6pdh* was increased by HFD, but SK alleviated the mRNA expression more effectively than SS. Therefore, it is postulated that the better anti-obesity effects of SK than SS could be related to the inhibition of lipogenesis via regulation of *Acc*, *Fas*, and *G6pdh* mRNA expression.

## 5. Conclusions

In conclusion, SS and SK improved the serum lipid profile, hepatic steatosis, and lipid accumulation in the adipose tissue of rats fed a high-fat diet. Notably, SK showed a better anti-obesity effect by reducing white adipose tissue mass compared to SS. Therefore, it is assumed that SK could be used as an effective dietary source to ameliorate obesity and dyslipidemia.

## Figures and Tables

**Table 1:** Composition of experimental diet.

	Groups	RD ^(1)^	HFD ^(2)^	HFD+SS	HFD+SK
Components					
Ingredient (g/kg)					
Casein		200.000	200.000	188.84	188.00
L-cystine		3.000	3.000	3.000	3.000
Corn starch		397.486	262.46	256.88	258.47
Dextrose		132.000	132.000	132.000	132.000
Sucrose		100.000	100.000	100.000	100.000
Cellulose		50.000	50.000	50.000	50.000
Lard			135.000	135.000	135.000
Soybean oil		70.000	70.000	67.930	66.280
Mineral mix ^(3)^		35.000	35.000	35.000	35.000
Vitamin mix ^(4)^		10.000	10.000	10.000	10.000
Choline chloride		2.500	2.500	2.500	2.500
*tert*-Butylhydroquinone		0.014	0.040	0.040	0.040
Steamed soybean				30.000	
Soybean *koji*					30.000
Total		1000.0	1000.0	1000.0	1000.0
Total energy (kcal)		4001.9	4674.8	4667.5	4665.5
Fat (kcal %)		15.8	39.5	39.2	39.2

^(1)^ RD: regular diet, ^(2)^ HFC: high-fat diet, ^(3)^ AIN-93-GX mineral mixture, and ^(4)^ AIN-93-VX vitamin mixture.

**Table 2:** RT-PCR primer sequences (5′ to 3′).

Transcript	Forward Primer	Reverse Primer
*Acc*	CAACGCCTTCACACCACCTT	AGCCCATTACTTCATCAAAGATCCT
*Fas*	GGAACTGAACGGCATTACTCG	CATGCCGTTATCAACTTGTCC
*G6pdh*	GTTTGGCAGCGGCAACTAA	GGCATCACCCTGGTACAACTC
*β-actin*	GTGGGGCGCCCCAGGCACCAGGGC	CTCCTTAATGTCACGCACGATTTC

**Table 3:** Proximate composition of steamed soybean and soybean *koji*.

Parameter (%)	Steamed Soybean	Soybean *Koji*
Moisture	1.7 ± 0.17 ***	3.5 ± 0.17
Carbohydrate	48.4 ± 2.96 *	39.9 ± 1.96
Crude protein	38.5 ± 0.9	39.9 ± 2.1
Crude fat	6.9 ± 0.44 ***	12.4 ± 0.61
Ash	4.5 ± 0.18	4.3 ± 0.11
Dietary fiber	37.3 ± 2.64	34.3 ± 1.29

* *p* < 0.05, *** *p* < 0.001.

**Table 4:** Free amino acid composition in steamed soybean and soybean *koji*.

Amino Acid (mg%)	Steamed Soybean	Soybean *Koji*
Urea	0.10 ± 0.01 ***	0.38 ± 0.01
Aspartic acid	0.09 ± 0.00 ***	<LLOQ ^(1)^
Threonine	0.26 ± 0.00 ***	0.49 ± 0.01
Serine	0.20 ± 0.01 **	0.1 ± 0.00
Asparagine	0.75 ± 0.02 **	0.6 ± 0.02
Glutamic acid	0.88 ± 0.03 ***	5.93 ± 0.03
α-aminoadipic acid	0.53 ± 0.03 ***	<LLOQ
Proline	0.72 ± 0.01 ***	<LLOQ
Glycine	0.18 ± 0.01 **	0.26 ± 0.01
Alanine	2.16 ± 0.06 **	1.29 ± 0.06
Citrulline	<LLOQ	0.18 ± 0.01
Valine	0.96 ± 0.04 ***	7.12 ± 0.27
Methionine	<LLOQ	0.74 ± 0.02
Isoleucine	0.54 ± 0.03 ***	3.24 ± 0.10
Leucine	0.69 ± 0.02 ***	10.6 ± 0.36
Tyrosine	1.15 ± 0.04 ***	5.07 ± 0.14
Phenylalanine	2.08 ± 0.07 ***	26.17 ± 0.73
β-alanine	0.29 ± 0.02 ***	<LLOQ
γ-amino-n-butyric acid	1.95 ± 0.04 ***	0.52 ± 0.01
Histidine	0.36 ± 0.01 ***	1.26 ± 0.02
Tryptophan	0.85 ± 0.03 ***	1.84 ± 0.09
Arginine	0.68 ± 0.07 **	<LLOQ
Total	15.42 ± 0.41 ***	65.8 ± 2.21

** *p* < 0.01, *** *p* < 0.001. ^(1)^ <LLOQ; lower limit of quantification.

**Table 5:** Fatty acid composition in steamed soybean and soybean *koji*.

Fatty Acid (% Total Fatty Acids)	Steamed Soybean	Soybean *Koji*
Myristic acid (C14:0)	0.15 ± 0.01	0.17 ± 0.02
Pentadecanoic acid (C15:0)	<LLOQ ^(1)^	0.49 ± 0.06
Palmitic acid (C16:0)	13.80 ± 0.76	16.03 ± 0.61
Heptadecanoic acid (C17:0)	0.76 ± 0.02 **	0.52 ± 0.04
Stearic acid (C18:0)	5.53 ± 0.05 **	6.40 ± 0.07
Arachidic acid (C20:0)	0.59 ± 0.02	0.55 ± 0.20
Heneicosanoic acid (C21:0)	22.05 ± 0.18 ***	10.72 ± 0.39
Behenic acid (C22:0)	0.61 ± 0.05	0.69 ± 0.03
Lignoceric acid (C24:0)	0.19 ± 0.01	0.22 ± 0.02
Saturated	43.67 ± 0.80 **	35.80 ± 0.27
Myristoleic acid (C14:1)	<LLOQ	0.16 ± 0.02
Palmitoleic acid (C16:1)	0.13 ± 0.01	0.16 ± 0.01
Oleic acid (C18:1n9c)	18.20 ± 0.07 **	20.39 ± 0.45
cis-11-Eicosenoic acid (C20:1)	0.36 ± 0.03	0.30 ± 0.01
Monounsaturated	18.69 ± 0.20 *	21.01 ± 0.77
Linolelaidic acid (C18:2n6t)	0.28 ± 0.20	<LLOQ
Linoleic acid (C18:2n6c)	29.01 ± 0.56 *	33.41 ± 0.88
cis-11,14-Eicosadienoic acid (C20:2)	0.09 ± 0.01 ***	<LLOQ
Linolenic acid (C18:3n3)	7.66 ± 0.18 **	9.67 ± 0.35
cis-5,8,11,14,17-Eicosapentaenoic acid (C20:5n3)	0.61 ± 0.05 ***	0.10 ± 0.02
Polyunsaturated	37.64 ± 0.67 **	43.19 ± 0.85

* *p* < 0.05, ** *p* < 0.01, *** *p* < 0.001. ^(1)^ <LLOQ; lower limit of quantification.

## Data Availability

The data presented in this study are available from the corresponding author upon request.
